# Bioactive Compounds of a Wheat Bran Oily Extract Obtained with Supercritical Carbon Dioxide †

**DOI:** 10.3390/foods9050625

**Published:** 2020-05-13

**Authors:** Sara Rebolleda, María Luisa González-San José, María Teresa Sanz, Sagrario Beltrán, Ángela G. Solaesa

**Affiliations:** 1 Department of Biotechnology and Food Science, University of Burgos, Plaza Misael Bañuelos s/n, 09001 Burgos, Spain; sarita155@hotmail.com (S.R.); marglez@ubu.es (M.L.G.-S.J.); tersanz@ubu.es (M.T.S.); 2 Department of Agriculture and Forestry Engineering, Food Technology, College of Agricultural and Forestry Engineering, University of Valladolid, 47011 Valladolid, Spain; angela.garcia.solaesa@uva.es

**Keywords:** wheat bran oily extract, alkylresorcinols, tocopherols, steryl ferulates, phenolic compounds, antioxidants, storage, supercritical fluid extraction

## Abstract

A wheat bran oily extract obtained with supercritical carbon dioxide at 25.0 ± 0.1 MPa and 40 ± 2 °C has been analyzed in order to determine some valuable bioactive compounds as alkylresorcinols, α-linolenic acid, steryl ferulates, tocopherols and phenolic compounds, which levels were around 47, 37, 18, 7 and 0.025 mg/g oily extract, respectively. To our knowledge, this is the first time that the presence of steryl ferulates has been observed in a supercritical fluid extract of wheat bran and that γ-tocopherol has been described in wheat bran oily extracts. Other common quality parameters, directly correlated with oxidative degradation, were also evaluated. Acidity values around 15% oleic acid were detected, while low levels of hydroperoxides (around 2.4 meq O_2_/kg) and very low levels of hexanal (0.21 ppb) were found. Composition of the wheat bran oily extract was stable during 155 days of storage at 21 °C and darkness, and only a slight decrease in alkylresorcinols and tocopherols contents (13% and 20%, respectively) was observed. These results indicated an attractive potential of the obtained oily extract for industrial applications as food ingredients, nutraceuticals, and others.

## 1. Introduction

The addition of antioxidants is the most widely used strategy for reducing oxidation, and the consequent loss of quality in food products. The food industry has been always interested in lipophilic antioxidants, and the most commonly used have been synthetic ones, such as butylated hydroxyanisole (BHA) and butylated hydroxytoluene (BHT). However, the often negative effects of these compounds on health and the increasing demand for natural additives by consumers highlight the necessity of new lipophilic antioxidants that could control oxidation during processing and storage [1]. Furthermore, natural antioxidants are also demanded by the food industry for inhibiting the enzymes that produce browning and hyperpigmentation, which occur in plants and animals by the synthesis of melanin and other brown pigments, as tyrosinase and other PPO (Poly-phenol-oxidase) enzymes. A wide variety of natural substances with a high phenolic content has been studied to determine their capacity as tyrosinase inhibitors and their potential to replace the most commonly used sulphiting agents, which are known to produce allergenic reactions, among other effects on human health [2].

Wheat bran is an important source of bioactive compounds, which are related to the human health-protective mechanisms of whole-grain cereals in general [3]. Some of these bioactive compounds, such as alkylresorcinols (AR), steryl ferulates and tocopherols are well known lipophilic or amphiphilic antioxidants, and all, and to a lesser extent phenolic compounds, can be extracted by supercritical fluid extraction processes, obtaining oily extracts enriched with them [4,5]. It is well-known that the solvent system used in the extraction process influences notably the composition and the quality of the oily extracts [6]. 

AR are a group of amphiphilic phenolic lipids present in many different organisms, such as plants, fungi and bacteria. AR are biomarkers for the presence of whole grain wheat and rye in food products and have been proposed for estimating the intake of whole grain products [7]. AR have a wide range of biological activities such as antibacterial, antifungal, antioxidant, enzyme inhibitor activities, each AR homologue having a different intensity of such activities [8], reason for which, determining the AR profile is of interest. Dietary AR have been also related to cancer prevention [9,10].

Wheat bran has been also reported to be an important source of steryl ferulates [11]. Steryl ferulates have been widely described for rice bran oil, and diverse health benefits, including hypocholesterolaemic–hypolipidaemic and anti-inflamatory activities have been associated with γ-oryzanol. Steryl ferulates are also considered as potent antioxidants due to the hydrogen-donating ability of the phenolic group of ferulic acid [12].

Cereals tocopherols are mainly located in bran and germ and wheat and rye have been described as the richest sources of tocopherols in the human diet. Tocopherols are usually employed as lipophilic antioxidants in food systems and “natural” tocopherols, isolated from natural sources have been described as more biologically active than their synthetic counterparts, although the bioactivity differs among homologues (α-, β-, γ- and δ) [13].

The healthy properties of whole wheat have been also associated to its phenolic content. The main phenolic compounds are hydroxycinnamic acids, as ferulic acids and hydroxybenzoic derivatives such as vanillic acids. Typically, in cereals, the phenolic compounds are present in free and bound forms linked to cell wall in the outer layers of caryopses [14]

The use of supercritical fluids as extraction agents is a well stablished technology with many industrial applications, mainly using supercritical carbon dioxide (scCO_2_) [15]. However, there is scarce published information about the characteristics of scCO_2_ wheat bran oily extracts. Therefore, new studies focused on collecting new information about this type of extracts, especially about the levels of diverse bioactive compounds, are necessary to evaluate their potential uses in the food industry.

The aim of this work was to evaluate the bioactive compounds (alkylresorcinol, steryl ferulates, tocopherols and phenolic compounds) contained in a scCO_2_ wheat bran oily extract (from now, SCWBOE). Furthermore, usual quality parameters and antioxidant capacity of the obtained extracts were also evaluated. Finally, the changes on the bioactive compounds, oil quality and global antioxidant capacity—during 155 days of storage at 21 ± 1 °C and in darkness—were assessed. 

## 2. Materials and Methods

### 2.1. Raw Material 

Oily extracts were obtained from wheat (*Triticum aestivum* L.) bran by supercritical CO_2_ extraction under previously optimized conditions [4,5]. Wheat bran was kindly provided by HASENOSA (Spain) and its humidity was (11% *w*/*w*). 

### 2.2. Supercritical Fluid Extraction (SFE) Equipment and Procedure 

The extraction experiments were carried out in a semi-pilot SFE-plant whose P&I diagram has been presented elsewhere [16]. The usual elements of an SFE-plant with solvent recycling were installed, i.e., pump, extractor, separator, heating and cooling systems and pressure dampers. Rupture disks and safety valves were installed for safety and the necessary instruments were installed for measurement and control of the process parameters.

In an SFE experiment, 300 g of wheat bran were placed in the extractor (2 L capacity) that was later pressurized with CO_2_ up to 25.0 ± 0.1 MPa. Then, the solvent was circulated through the extractor at 40 ± 2 °C, with a solvent flow of 8 ± 1 kg CO_2_/h and during an extraction time of 120 min in order to circulate enough amount of scCO_2_ for completing the extraction of the soluble compounds. The solvent was continuously recycled to the extractor after removing the solute in the separator that was kept at 4.9 ± 0.6 MPa and 24 ± 2 °C. Co-extracted water was removed by centrifugation at 12,857× *g* during 30 min.

Three different extracts were obtained and evaluated. The average extraction yield obtained was 2.5 ± 0.1 g extract/100 g dry bran.

### 2.3. Chemicals

Fatty acid methyl esters, AR (C15, C17, C19 and C25), phenolic (ferulic, vanillic and syringic acids, vanillin and *p*-OH-benzaldehyde), tocopherol standards (α, β, δ and γ), 2,2′-azino-bis (3-ethylbenzothiazoline-6-sulfonic acid) diammonium salt (ABTS), 6-Hydroxy-2,5,7,8-tetramethylchromane-2-carboxylic acid (Trolox), 2,2-diphenyl-1-picryhydrazyl (DPPH) and 2,4,6-tri(2-pyridyl)-*s*-triazine (TPTZ), were purchased from Sigma–Aldrich Co. (St. Louis, MO, USA). Syringic aldehyde was supplied by Extrasynthese (Genay, France), methyl tricosanoate by Larodan (Malmö, Sweden) and K_2_O_8_S_2_, FeCl_3_ and FeSO_4_ were purchased from Panreac (Barcelona, Spain).

### 2.4. Analytical Methods

#### 2.4.1. Methods for Quantification of Different Bioactive Compounds

##### Fatty Acids Content and Profile

The fatty acids profile was determined by the AOAC official method [17]. Briefly, the fatty acid methyl esters were first prepared and then analyzed by gas chromatography (Agilent Technologies, Santa Clara, CA, USA). Split injection (50:1) and a flame ionization detector (FID), both at 250 °C, were used. The separation was carried out with helium (1.8 mL/min) as carrier gas in a fused silica capillary column (OmegawaxTM-320, 30 m × 0.32 mm i.d.). The column temperature was programmed starting at a constant temperature of 180 °C for 20 min, heated to 200 °C at 1 °C/min, held at 200 °C for 1 min, heated again to 220 °C at 5 °C/min and finally held at 220 °C for 20 min. Fatty acid methyl esters were identified by comparison of their retention times with those of chromatographic standards. Their quantification was performed using response factors obtained with their chromatographic standards and methyl tricosanoate as internal standard.

##### Alkylresorcinols Content and Profile

Alkylresorcinols were determined by HPLC-DAD (Agilent Technologies, Santa Clara, CA, USA) according to a previously reported method [4]. The column used was a Kromasil C18-5 (250 × 4.6 mm) operated at 25 °C. The mobile phase was methanol (A) and water (B) and the following gradient was used: 2% B to 0% B in 10 min. The total run time was 50 min. The injection volume was 100 μL of methanolic solutions of SCWBOE (10 mg/mL). All AR were monitored at 280 nm at a flow rate of 0.6 mL/min. AR were identified by comparing their retention time and UV-Vis spectrum with the corresponding standards.

##### Steryl Ferulates Content and Profile

Steryl ferulates were analyzed by HPLC-DAD (Agilent Technologies, Santa Clara, CA, USA). Separation was carried out in a Zorbax XDB C18 column (150 × 4.6 mm, 5 µm) using isocratic elution with acetonitrile/methanol/isopropanol (50:40:10). Methanolic solutions of the SCWBOE (10 mg/mL) were injected (30 µL). Steryl ferulates were monitored at 330 nm, at a flow rate of 1 mL/min, and identified using a standard mixture of steryl ferulates and literature data [18].

##### Tocopherols Content and Profile

Solid phase extraction (SPE) followed by HPLC-DAD (Agilent Technologies, Santa Clara, CA, USA) was used for the determination of tocopherols in wheat bran oily extracts according to a previously published methodology [19]. In a first step, tocopherols were extracted in silica cartridges (1000 mg/6 mL, Sep-Pak^®^, Waters, Spain) that were previously conditioned (5 mL *n*-hexane), charged with 1 mL of SCWBOE methanolic solution (0.1 g/mL *n*-hexane) and equilibrated (5 mL *n*-hexane). Elution was performed with 5 mL of *n*-hexane–diethyl-ether (99:1, *v*/*v*) and 50 mL of *n*-hexane–diethyl-ether (99:2, *v*/*v*). The collected fraction was evaporated under reduced pressure at 45 °C and the dry residue obtained was dissolved in 1.5 mL of *n*-hexane. In a second step, 50 µL of this solution were injected in a HPLC. The column used was an ACE 5 silica column (250 mm × 4.6 mm) and the mobile phase was hexane and 2-propanol (99:1) at a flow rate of 1 mL/min for 15 min. Tocopherols were identified and quantified at 296 nm by using calibration curves obtained with the corresponding standard compounds.

##### Phenolic Compounds Content and Profile

Phenolic compounds were extracted from SCWBOE previous to their analysis by HPLC-DAD (Agilent Technologies, Santa Clara, CA, USA). Then, 2.0 g of SCWBOE was extracted twice with 2 mL of methanol by vortex agitation for 2 min. The two methanol extracts were mixed and centrifuged at 3214× *g* for 30 min, the supernatant was separated and evaporated under vacuum at 40 °C. The dry residue was suspended in 2 mL of water: methanol (80:20), filtered (20 µm) and analyzed by a HPLC-DAD system according to the method previously reported by Pérez-Magariño et al. [20] Chromatographic separation was performed in a Spherisorb ODS2-3µm column (250 × 4.6 mm) at a flow rate of 0.6 mL/min with (A) water/acetic acid (98:2) and (B) water/acetonitrile/acetic acid (78:20:2) and the following linear gradient: from 0% to 25% solvent B in 25 min, from 25% to 70% B in 35 min, from 70% to 100% B in 40 min and then isocratic for 20 min. Diode array detection was performed from 200 to 400 nm. The injection volume was 200 µL. The phenolic compounds analyzed were identified by comparing their retention times and UV-Vis spectra with their respective standard according to previously published data [20]. Quantification was performed by using the calibration curves obtained with the corresponding standard compound.

#### 2.4.2. Determination of Some Usual Oil Quality Parameters

##### Acidity Value (AV)

A modification of the AOCS Ca 5a-40 method [21] was used to evaluate the SCWBOE acidity by automatic titration with potassium hydroxide solution (Metrohm 905 Titrando, Herisau, Switzerland) using a pH electrode (Solvotrode, Metrohm, Herisau, Switzerland). Results were given as percentage of oleic acid (*w*/*w*).

##### Peroxide Value (PV)

Peroxide value was determined potentiometrically according to a modification of the AOCS Cd 8-53 method [21] by titration with sodium thiosulfate using an automatic titrator (Metrohm 905 Titrando, Herisau, Switzerland) equipped with a platinum electrode (Combined LL Pt-ring electrode, Metrohm, Herisau, Switzerland). Results were expressed in oxygen milliequivalents per kg of SCWBOE (meq O_2_/kg).

##### Hexanal Content

Hexanal concentration was analyzed by GC–MS after solid phase dynamic extraction (SPDE) of the sample headspace (HS). A coated SPDE-syringe with a non-polar 90% polydimethylsiloxane and 10% activated carbon sorbent (Chromtech, Idstein, Germany) was used in the HS-SPDE autosampler (CTC CombiPalautosampler, CTC Analytics, Switzerland). A pre-equilibration step of 1 min at 70 °C was carried out. The coated needle was connected to a 2.5 mL gastight syringe and 50 extraction cycles of 1000 µL each, at a speed of 40 µL/s, were carried out. For the compounds desorption and injection, 500 µL of helium was pulled into the SPDE-syringe over 30 s, and then pumped into the GC inlet at 15 µL/s. GC-MS analyses were performed using a gas chromatograph (Agilent Technologies 6890N, Network GC system) coupled to a mass selective spectrophotometer detector (Agilent Technologies, model 5973 inert) and an Enhanced Chemstation version D.01.02.16 software (Agilent Technologies, Santa Clara, CA, USA). Separation was carried out in a capillary column (Carbowax20M, 60 m × 0.32 mm i.d., Quadrex Corporation, New Haven, CT, USA) with helium at a constant flow of 1.0 mL/min. The injector temperature was 250 °C and splitless injection mode was used. The initial oven temperature was 40 °C and it was increased to 240 °C at 3 °C/min. The mass spectrophotometer was set in electron-impact (EI) mode at 70 eV with a voltage multiplier of 1835 V.

#### 2.4.3. Total Antioxidant Capacity of the Wheat Bran Oily Extract

The antioxidant profile of SCWBOE was evaluated considering different usual antioxidant assays, which give complementary information, allowing to obtain an antioxidant profile of the products under study [22]

##### FRAP Assay

FRAP assays were selected to evaluate the reducing power of SCWBOE [4]. The reaction takes place by mixing 30 μL of an ethanol solution of SCWBOE (5 mg/mL) and 970 µL of FRAP reagent. The FRAP reagent was prepared with 25 mL of 0.3 M sodium acetate buffer solution at pH 3.6, 2.5 mL of 10 mM TPTZ (tripyridyl-S-triazine), 2.5 mL of FeCl_3_ (20 mM), and 3 mL of milli-Q water. The reaction was carried out at 37 °C for 30 min and the absorbance was measured at 593 nm (Hitachi U-2000 spectrophotometer, Tokyo, Japan). FeSO_4_ was used for calibration and the reductive power of the oily extract was expressed as µmol Fe (II)/g SCWBOE.

##### ABTS Assay

ABTS assay was selected by its capacity to evaluate both lipophilic and hydrophilic antioxidants. The radical ABTS•+ was generated by mixing 7 mM solution of ABTS in water with 2.45 mM K_2_O_8_S_2_ (1:1) and held in darkness during 16 h [5]. The ABTS^•+^ antioxidant reaction mixture contained 20 µL of SCWBOE diluted in ethanol (5 mg/mL) and 980 µL of radical ABTS•+. Absorbance was measured at 734 nm (Hitachi U-2000 spectrophotometer, Tokyo, Japan) after 20 min of reaction. Trolox was used as antioxidant standard. Results were expressed as µmol Trolox/g SCWBOE.

##### DPPH Assay

DPPH assay was selected by its capacity to evaluate lipophilic antioxidant. Next, 20 µL of SCWBOE diluted in ethanol (5 mg/mL) was mixed with 980 µL of DPPH• (2,2-diphenyl-1-picrylhydrazyl radical) solution (50.7 µM) and the absorbance at 517 nm was measured (Hitachi U-2000 spectrophotometer, Tokyo, Japan) after 60 min of reaction at ambient temperature and darkness [4]. Methanolic solutions of known Trolox concentrations were used for calibration. Results were expressed as µmol Trolox/g SCWBOE.

#### 2.4.4. Evolution of Wheat Bran Oily Extract Composition during Storage

SCWBOE was stored at 21 ± 1 °C and in darkness for 155 days. The following parameters were monitored over that time: AR and tocopherols, AV, PV, hexanal content and ABTS values.

### 2.5. Statistical Analysis

All the determinations were conducted in three different extracts, and results were expressed as mean ± standard deviation (SD). Differences between data means were compared by least significant differences (LSD) calculated using STATGRAPHICS Centurion XVI.I.

## 3. Results and Discussion

### 3.1. Bioactive Compounds Evaluated in the Obtained Supercritical Wheat Bran Oily Extracts

Different bioactive compounds of interest to the food industry—such as alkylresorcinols, tocopherols, steryl ferulates, phenolic compounds and polyunsaturated fatty acids (PUFA)—were found in the obtained SCWBOE (Table 1). 

The fatty acid profile of SCWBOE revealed that the polyunsaturated fatty acids (PUFA) level was around 63% of the total extracted fatty acids, while saturated fatty acids were around 18%. Linoleic acid (LA, C18:2ω6) was the major PUFA detected (around 58% of total fatty acids), and significant quantities of α-linolenic acid (ALA, C18:3ω3) were also quantified. Both compounds are essential PUFA, precursors of the omega-6 and omega-3 families, respectively, and therefore, very important in the human diet. The large PUFA content of SCWBOE indicated the high nutritional value of this product, better than some of the commonly used oils, which have low levels of PUFA (e.g., palm oil, with around 10% PUFA on average) and often show very low levels of ALA (e.g., sunflower, sesame, and palm oils, with around 0.5% of total fatty acids on average) [23].

Information on the AR profile could be useful for decision making in terms of their utility to the food industry, since different intensities of the biological activity of each AR homologue have been reported [8]. The AR profile of SCWBOE (Table 1) was similar to that previously reported for wheat bran [24], with C19 and C21 homologues being the major ones, with levels of around 30% and 48% of the total extracted AR, respectively. The total amount of AR in the SCWBOE obtained in this work (117 mg/100 g dry bran) was higher that the obtained by Athukorala et al. [25] using a two-step sequential scCO_2_ extraction technique, without and with ethanol, for AR extraction from commercial wheat bran. The AR were extracted (68 mg/100 g wheat bran) in the second step, when using ethanol-modified scCO_2_. It was also much higher than the reported for oils obtained from wheat germ by aqueous enzymatic extraction (1.5 ± 0.6 mg/g oil) or via ethyl acetate extraction (1.50 ± 0.04 mg/g oil) [26].

Significant levels of steryl ferulates were found in SCWBOE (Table 1). The steryl ferulate profile of SCWBOE (54% to campestanyl + sitosteryl ferulates, 32% to sitostanyl ferulate and 13% campesteryl ferulate) was similar to the reported in acetone extracts of wheat bran [27]; however, steryl ferulate levels obtained in this work (18 ± 1 mg/g) were much higher than those previously described for hexane extracted oils (3.1 mg/g) [28]. Similar results were reported for rice bran oily extracts, where γ-oryzanol (main rice steryl ferulate) yield in extracts obtained with scCO_2_ (17.6 ± 0.1 mg/g) [29] was approximately four times higher than the highest yield obtained by extraction with liquid organic solvents [30]. Some authors pointed out that wheat bran steryl ferulates have good antioxidant activity, even more than rice bran steryl ferulates [12]. Steryl ferulates have been widely described for rice bran oil, and diverse health benefits, including hypocholesterolaemic–hypolipidaemic and anti-inflamatory activities, have been associated with γ-oryzanol [30]. Steryl ferulates are also considered as potent antioxidants due to the hydrogen-donating ability of the phenolic group of ferulic acid [12]. The occurrence of ferulic acid esters of sterols in wheat grain and bran has been previously reported [18,27,28]; however, to our knowledge, this paper is the first that describes the presence of steryl ferulates in a supercritical extract of wheat bran.

The mean tocopherol content of SCWBOE was around 7 mg/g, (Table 1), which is a much higher value than those described for other vegetable oils such as crude soybean oil (1.4 mg/g) [23] or that obtained by scCO_2_ extraction of rice bran (2.14 ± 0.28 mg/g) [29], being more similar to the global level described for a wheat bran oil, also obtained by SFE, from *Triticum durum* variety (4.3 ± 0.7 mg/g oil) [31]. The variability of tocopherol composition among the different wheat varieties [32], together with the effect of the extraction conditions [5] could explain the quantitative differences of tocopherol content described in each paper. Besides, to our knowledge, this paper quantifies four different tocopherol isomers in an SCWBOE for the first time. This was possible because a normal phase chromatographic method, which enables the separation of β- and γ-tocopherols, was used. When reverse-phase columns were used, this separation was not possible [1]. The α-tocopherol proportion (57%) in SCWBOE was similar to that described for wheat germ and cottonseed oils, and the γ-tocopherol proportion (39%) was higher than that reported for sunflower and olive oils [33]. These results could be of interest to the food industry due to the high α-tocopherol biological activity and the large effectiveness of γ-tocopherol to inhibit the oxidation of fats and oils [1]; although it should be noted that the antioxidant activity of each tocopherol homologue depends, among other factors, on the food matrix in which they are incorporated [34].

Wheat bran has been described as a rich source of phenolic compounds [3]; however, due to the low solubility of these compounds in scCO_2_ [35], low phenol content was found in the SCWBOE obtained in this work (Table 1). The total phenolic compounds content (0.025 mg/g) was found to be significantly lower than the reported for phenolic rich oils such as olive oil, which polyphenol content usually varies between 0.1 and 0.3 mg/g [23]. Previous works evaluated the global polyphenol content of supercritical wheat bran oil; however, individual phenolic compounds have not been previously evaluated. Some of the main phenolic acids reported in wheat bran are ferulic, vanillic and syringic acids [36]. The solubility of these acids in scCO_2_ follows the trend vanillic acid > ferulic acid > syringic acid [35]. These solubility differences may explain the predominance of vanillic acid in the studied SCWBOE. Furthermore, the higher solubility of aldehydes in scCO_2_ regarding that of their corresponding acids explained the high level of vanillin and the presence of other aldehydes. Moreover, the absence of other phenolic acids such as p-cumaric and caffeic acids, also reported in wheat bran [36], could be due to their low solubility in scCO_2_ [35].

### 3.2. Common Quality Parameters Evaluated in the Obtained Supercritical Wheat Bran Oily Extracts

Common parameters used to evaluate oil quality were considered as an index of the SCWBOE quality. Since triglycerides hydrolysis and lipid oxidation have been described as the main deterioration factors of edible oils, different parameters directly associated with these degradative processes have been evaluated. Triglycerides hydrolysis releases free fatty acids (FFA) susceptible to oxidation. Lipid oxidation occurs in two steps—the first is the primary oxidation which is correlated to the oxidation of FFA and gives unstable hydroperoxides, susceptible of decomposition during the secondary oxidation, in which a complex mixture of volatile, non-volatile, and polymeric products is formed. Secondary oxidation products include aldehydes, ketones, alcohols, etc., being hexanal one of them. Therefore, the Acidity Value (AV), which is related with the free fatty acid content, and the Peroxide Value (PV) and the hexanal content, which are respectively related to the primary and secondary oxidation products, were chosen to evaluate SCWBOE quality. 

SCWBOE was found to have a relatively large quantity of FFA, with an AV of around 15% oleic acid (Figure 3). This is not desirable, because FFA are highly prone to oxidation with the consequent decrease in the quality and stability of oily products. The large values obtained could be due to the possible wheat bran lipid hydrolysis during storage, but also, some hydrolysis could have occurred during SFE. It is well-known that wheat lipase activity is present mainly in the bran fraction and bran triacylglycerides are substantial substrates to lipases. Physical damages occurring during wheat milling enhance the contact between enzymes and substrates, favouring the hydrolysis of triglycerides. Furthermore, the bran humidity (around 11%) is high enough as to allow lipases action. Therefore, minimization of this type of hydrolysis should be avoided by improving wheat bran storage conditions. Furthermore, the AV of wheat bran oily extracts could be reduced by performing SFE with on-line fractionation of the extract, or else though a refining process [37].

The PV of the SCWBOE obtained in this work indicated a low level of hydroperoxides (around 2.4 meq O_2_/kg) (Figure 3). The absence of oxygen during SFE has been correlated with lower PV of SFE oily extracts compared with the oily extracts obtained by other extraction techniques [38], although, in contrast, Jung et al. [39] reported higher PV (> 20 meq O_2_/kg) in SFE than in hexane extracted wheat oily extracts. However, these authors. [39] also noted a lower AV (around 2.5 mg KOH/g). Both results indicated a strong oxidative degradation of free fatty acids, and consequently the levels of FFA decrease as much as PV levels increase. However, the low PV obtained in this work indicated a low oxidative degradation of the FFA, which remained intact, resulting in the high AV detected. The drying and grinding steps used by Jung et al. [39] could explain the intense oxidation detected in the oily extracts they studied. It is also important to consider the lower levels of antioxidants found in oily extracts obtained from dry wheat bran than from non-dried wheat bran [5].

The hexanal content is an important parameter to evaluate the oxidative deterioration of linoleic acid-containing oily extracts and it has been related to the perception of rancidity in sensorial evaluation [40]. The hexanal content of the SCWBOE analyzed right after being obtained was around 0.21 ppb (Figure 3), which is far below the odour and flavour threshold values reported for hexanal in oil: 31 and 150 ppb, respectively [41].

### 3.3. Evaluation of Antioxidant Capacity of the Obtained Supercritical Wheat Bran Oily Extracts

The antioxidant capacity of the SCWBOE was evaluated as a hypothetical and theoretical index of the antioxidant stability of the obtained oily extracts and also as a theoretical index of their potential as natural antioxidant additives or food ingredients. The results obtained indicated that the SCWBOE had considerable antioxidant capacities (Table 2). The antioxidant activity evaluated by the DPPH method was higher than that reported by Durante et al. [31] (1.90 µmol Trolox/g) for supercritical extracted wheat bran oily extracts, which had lower levels of tocopherols, strong lipophilic antioxidants, than those detected in the oily extract obtained in this study. Similarly, the antioxidant capacity evaluated by the ABTS method (270 µmol Trolox/g) was higher than that reported for other vegetable oils such as olive and sunflower oils (12.8 and 2.4 µmol Trolox/mL, respectively) [42]. These differences could be explained considering the different antioxidant composition of the different products. The oily extracts under study in this work had higher levels of tocopherols than those reported for olive (0.1–0.3 mg/g) and crude sunflower oils (0.6–0.7 mg/g) [23] Furthermore, alkylresorcinols and steryl ferulates, which are also strong antioxidants, are not present in olive and sunflower oils.

### 3.4. Evolution of the Obtained Supercritical Wheat Bran Oily Extracts during Storage

The food industry is interested in fatty food ingredients and oils stable over time; thus, lipid oxidation is the most important parameter to be considered. For that reason, parameters correlated with lipid oxidation, antioxidants (AR and tocopherol), oxidative stage index (AV, PV and hexanal) and global antioxidant capacity (ABTS) of the obtained oily extracts were evaluated, at different time intervals, during storage at 21 °C and in darkness. 

**Figure 1:**
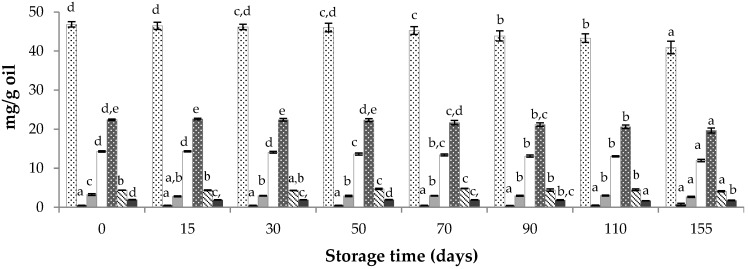
Variation of alkylresorcinol (AR) contents during storage at 21 ± 1 °C and darkness of the SCWBOE. Different letters within the same compound indicate significant mean differences according to LSD test at *p* < 0.05. 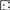 Total AR, 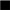 C15-AR, 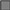 C17-AR, 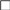 C19-AR, 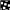 C21-AR, 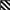 C23-AR, 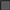 C25-AR.

Regarding the content of antioxidants, results indicated that the levels of total AR remained about constant until the second month of storage, but some statistically significant differences were found after 90 days of storage (Figure 1). Global losses of AR around 13% were obtained after 155 days of storage. C17 and C19 AR presented the higher degradation ratios—around 18% and 16%, respectively—while C23 decreased around 7% and C15 around 4%. No relationship between the chain length and the losses of each AR was found. 

**Figure 2:**
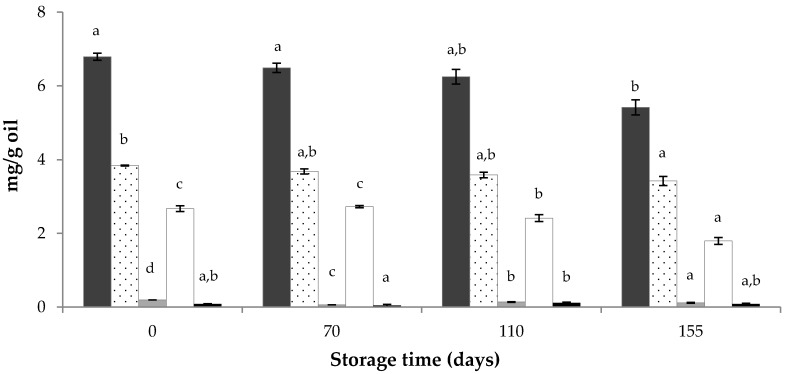
Changes of tocopherol levels during storage at 21 ± 1 °C and darkness of the SCWBOE. Different letters within the same compound indicate significant mean differences according to LSD test at *p* < 0.05. 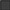 Total tocopherol, 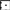
α-tocopherol, 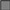 β-tocopherol, 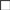 γ-tocopherol, 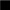 δ-tocopherol.

Similarly, qualitative and quantitative losses of tocopherols were also observed (Figure 2), and they were more intense for γ-tocopherol (33%) than for α-tocopherol (11%) at the end of the storage time. These results are similar to those described for olive oils stored at 20 °C and darkness [43]. These authors found reductions ranging from 6% to 9% for α-tocopherol, and from 35% to 44% for β+γ-tocopherol, after 180 days of storage.

**Figure 3:**
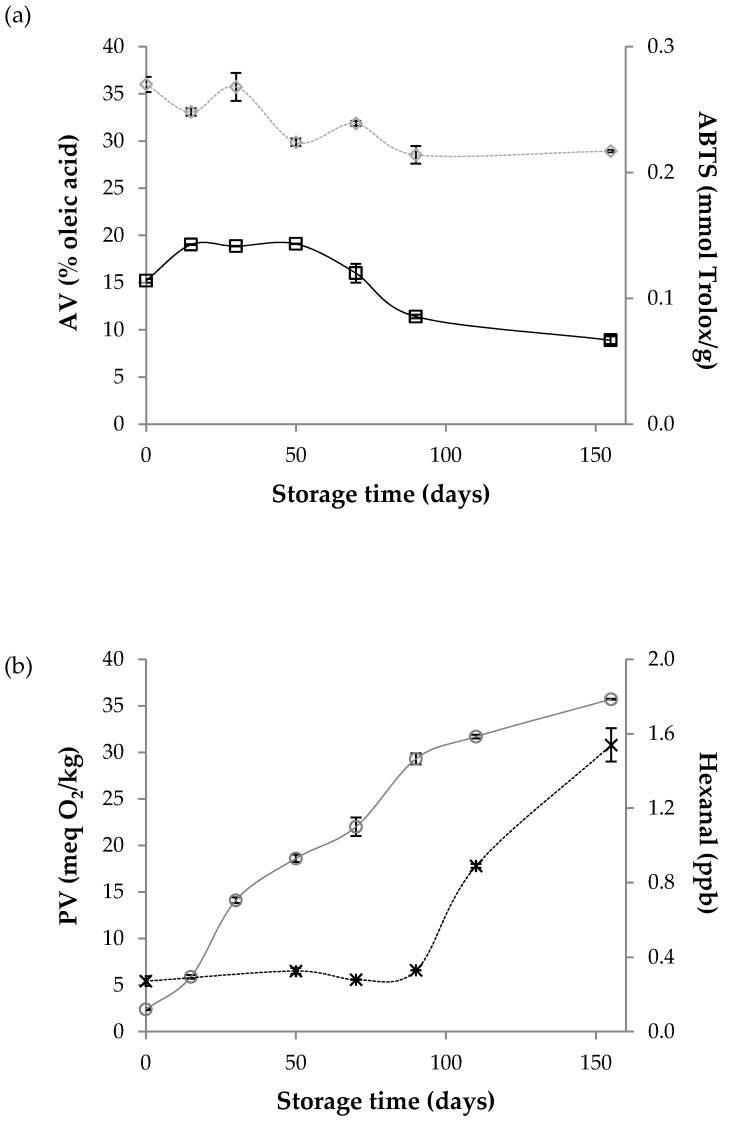
Evolution of: (**a**) ☐ acidity value (AV) and ◇ antioxidant activity (ABTS values) and (**b**) ○ peroxide value (PV) and ✕ hexanal content, of the SCWBOE, measured during a storage period of 155 days at 21 ± 1 °C and in darkness.

The decrease in tocopherols, but mainly the decrease in γ-tocopherol was well correlated with the AV evolution. While γ-tocopherol levels remained constant, AVs were also constant (until the second month of storage, Figure 3), and when γ-tocopherol began to decrease significantly, a decrease in AV values was observed, together with the increase in hexanal values. 

Regardless the strong antioxidant potential of tocopherols and other antioxidants such as AR, total inhibition of the oxidation of FFA was not possible and, for that reason, PV increased from the first month of storage. The more peroxides were generated, the more tocopherols and AR were degraded to block the formed peroxides (antioxidant protection), although the total blocking was not produced, resulting enough peroxides accumulation to allow the development of the secondary oxidation steps, which began to be predominant after the third month of storage, when the hexanal content started growing (Figure 3). Despite this process, the hexanal content remained below the odour and flavour threshold value of hexanal [41], and also far below the limit reported for the perception of rancidity in other oils (1 mg/kg) [40].

The evolution of the global antioxidant capacity monitored by the ABTS assay was well correlated with the progressive loss of antioxidant compounds. In any case, after the storage period evaluated, the SCWBOE retained significant global antioxidant activity, higher than the antioxidant activity reported for different essential oils [44] and also for other vegetable oils, such as olive and sunflower oils [42].

## 4. Conclusions

Extraction using supercritical CO_2_ allowed us to obtain oily extracts from wheat bran with a high content of valuable bioactive compounds such as alkylresorcinols, steryl ferulates, tocopherols, and a small amount of other phenolic compounds, which made the extracts have low levels of oxidation parameters (low hydroperoxides and hexanal content) and relatively high antioxidant global capacity. These oily extracts also had a good stability during storage, which suggests that this type of oily product could be an interesting food ingredient or a natural antioxidant additive able to prevent fat oxidation, although this should be contrasted with future studies. However, the level of free fatty acids found in the SFE wheat bran oily extract obtained in this work indicates that the storage conditions of wheat bran should be well controlled in order to avoid—or, at least, minimize—its oxidation process; otherwise, the oily extracts should undergo a refining process. 

## Figures and Tables

**Table 1:** Bioactive compounds evaluated in the SCWBOE.

Palmitic acid (C16:0)		118 ± 2
Stearic acid (C18:0)		7.9 ± 0.1
Oleic acid (C18:1)		114 ± 3
Linoleic acid (C18:2)		410 ± 10
α- linolenic acid (C18:3)		37.3 ± 0.8
Other fatty acids		25 ± 4
Total fatty acids (mg/g SCWBOE)		712 ± 20
C15- alkylresorcinol (R_1_=C_15_H_31_)	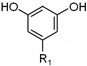	0.52 ± 0.01
C17- alkylresorcinol (R_1_=C_17_H_35_)		3.3 ± 0.2
C19- alkylresorcinol (R_1_=C_19_H_39_)		14.3 ± 0.3
C21- alkylresorcinol (R_1_=C_21_H_43_)		22.4 ± 0.2
C23- alkylresorcinol (R_1_=C_23_H_47_)		4.40 ± 0.03
C25- alkylresorcinol (R_1_=C_25_H_51_)		1.97 ± 0.06
Total alkylresorcinols (mg/g SCWBOE)		46.9 ± 0.8
Campesteryl ferulate (R_1_=H)	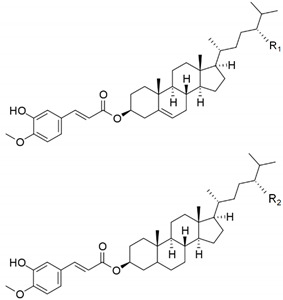	2.4 ± 0.1
Sitosteryl ferulate (R_1_=CH_3_) + Campestanyl ferulate (R_2_=H)		9.9 ± 0.5
Sitostanyl ferulate (R_2_=CH_3_)		5.9 ± 0.4
Total steryl ferulates (mg/g SCWBOE)		18 ± 1
α- Tocopherol (R_1_=R_2_=R_3_=CH_3_)	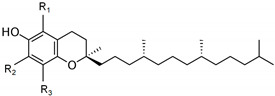	3.84 ± 0.01
β- Tocopherol (R_1_= R_3_=CH_3_; R_2_=H)		0.20 ± 0.01
γ- Tocopherol (R_1_=H; R_2_=R_3_=CH_3_)		2.67 ± 0.08
δ- Tocopherol(R_1_= R_2_=H, R_3_=CH_3_)		0.09 ± 0.01
Total tocopherols (mg/g SCWBOE)		6.8 ± 0.1
Vanillin (R_1_=H; R_2_=CHO; R_3_=OCH_3_)	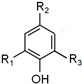	13.8 ± 0.1
Vanillic acid (R_1_=H; R_2_=COOH; R_3_=OCH_3_)		3.5 ± 0.5
Syringic aldehyde (R_1_=R_3_=OCH_3_; R_2_=CHO)		3.4 ± 0.8
Ferulic acid (R_1_=H; R_2_=(CH)_2_COOH; R_3_=OCH_3_)		1.8 ± 0.3
Syringic acid (R_1_=R_3_=OCH_3_; R_2_=COOH)		1.6 ± 0.4
*p-*Hydroxybenzaldehyde (R_1_=R_3_=H; R_2_=CHO)		0.7 ± 0.1
Phenolic compounds (mg/kg SCWBOE)		25 ± 2

Values are mean ± standard deviation of three extracts (*n* = 3).

**Table 2:** Antioxidant profile of the SCWBOE.

Antioxidant Method	Antioxidant Mechanism	Units	Value
DPPH	SET	µmol Trolox/g SCWBOE	26 ± 2
FRAP	SET	µmol Fe (II)/g SCWBOE	228 ± 12
ABTS	SET/HAT	µmol Trolox/g SCWBOE	270 ± 6
